# Ultrafine Particles from Traffic Emissions and Children’s Health (UPTECH) in Brisbane, Queensland (Australia): Study Design and Implementation

**DOI:** 10.3390/ijerph120201687

**Published:** 2015-02-02

**Authors:** Wafaa Nabil Ezz, Mandana Mazaheri, Paul Robinson, Graham R. Johnson, Samuel Clifford, Congrong He, Lidia Morawska, Guy B. Marks

**Affiliations:** 1 Woolcock Institute of Medical Research, 431 Glebe Point Road, Glebe, NSW 2037, Australia; E-Mails: dr.pdrobinson@googlemail.com (P.R.); guy.marks@sydney.edu.au (G.B.M.); 2 International Laboratory for Air Quality and Health, Institute of Health and Biomedical Innovation, Queensland University of Technology, 2 George Street, Brisbane, QLD 4000, Australia; E-Mails: m.mazaheri@qut.edu.au (M.M.); g.johnson@qut.edu.au (G.R.J.); samuel.clifford@qut.edu.au (S.C.); c.he@qut.edu.au; (C.H.) l.morawska@qut.edu.au (L.M.); 3 South Western Sydney Clinical School, University of New South Wales, Sydney, NSW 2052, Australia

**Keywords:** ultrafine particles, children, traffic, respiratory, air pollution, monitoring

## Abstract

Ultrafine particles are particles that are less than 0.1 micrometres (µm) in diameter. Due to their very small size they can penetrate deep into the lungs, and potentially cause more damage than larger particles. The Ultrafine Particles from Traffic Emissions and Children’s Health (UPTECH) study is the first Australian epidemiological study to assess the health effects of ultrafine particles on children’s health in general and peripheral airways in particular. The study is being conducted in Brisbane, Australia. Continuous indoor and outdoor air pollution monitoring was conducted within each of the twenty five participating school campuses to measure particulate matter, including in the ultrafine size range, and gases. Respiratory health effects were evaluated by conducting the following tests on participating children at each school: spirometry, forced oscillation technique (FOT) and multiple breath nitrogen washout test (MBNW) (to assess airway function), fraction of exhaled nitric oxide (FeNO, to assess airway inflammation), blood cotinine levels (to assess exposure to second-hand tobacco smoke), and serum C-reactive protein (CRP) levels (to measure systemic inflammation). A pilot study was conducted prior to commencing the main study to assess the feasibility and reliably of measurement of some of the clinical tests that have been proposed for the main study. Air pollutant exposure measurements were not included in the pilot study.

## 1. Introduction

Ultrafine particles (UFP) are particles that are less than 0.1 micrometres (µm) in diameter. Compared to larger particles, UFP have a larger relative surface area. This may be associated with greater toxicity, compared to larger particles, because their smaller diameter means they are more likely to deposit in the lung parenchyma and their greater surface area means they are potentially more reactive [1].

Emissions generated by fossil fuel combustion by motor vehicles are a major source of UFP [2]. The World Health Organization (WHO) has concluded, based on a review of toxicological evidence, that it is likely that UFP do have adverse effects on human health [3]. However, the existing body of epidemiological evidence is insufficient to determine an exposure-response relationship. Consequently, there are currently no health-based guidelines recommending threshold concentrations of UFP that should not be exceeded [1] Some previous studies have not allowed separate estimation of the specific health effects of UFP, apart from the effect of larger particle fractions, such as those measured as PM_2.5_ and PM_10_ [2]. One study that looked at the effects of UFP on cyclists found a non-significant association with increased exhaled nitric oxide and decreased lung function [4], another study found no association with UFP and hospital admission for asthma but a significant association with larger particles and other pollutants [5], while a third study in 1997 found that decrease in peak expiratory flow among children was associated with PM_10_ rather than UFP [6], other studies have found health effects mainly in people with asthma [7,8]. 

The motivation for the Ultrafine Particles from Traffic Emissions and Children’s Health (UPTECH) study is to provide epidemiological evidence about the effects of traffic-related UFP on human respiratory health, in general, and children’s respiratory health, in particular. The main hypothesis was that, among children aged 8 to 11 years, variation in long-term exposure to UFP is associated with variation in certain health states and that this association is independent of the effects of other factors including other air pollutants, housing conditions and indoor exposures, and socio-economic factors. 

The health states hypothesised to be related to spatial variation in UFP concentration included respiratory symptoms (wheeze and cough), spirometric and peripheral airway function, airway inflammation and systemic inflammation. This paper describes the methods used in the UPTECH study and presents the findings of a pilot study that was performed to evaluate some aspects of the study methodology. 

## 2. Design and Methods 

### 2.1. Overview of the Cross-Sectional Study Design

We have conducted a cross-sectional study to examine the association between spatial variation in exposure to UFP and spatial variation in the occurrence of respiratory symptoms, illnesses and impaired function. By choosing schools as the exposure site, we are able to capture children’s daytime exposure to UFP, which includes a large proportion of their overall exposure. Although this exposure is assigned to children at the level of their school, outcomes and covariates, including pollutant exposures at home and during travel to and from school, were measured and assigned individually [9]. 

The main study is a cross-sectional study that was conducted in 25 primary schools in the Brisbane Metropolitan Area of Queensland, Australia, between October 2010 and August 2012 The long term average maximum temperatures in Brisbane are 30 °C in summer and 22 °C in winter. A pilot study was conducted in two schools in March 2009 (early autumn) to evaluate some of the study measurement tools. The long term average maximum temperature for Brisbane in March is 29 °C.

### 2.2. Ethics Approvals

The study was approved by the Queensland University of Technology Human Research Ethic Committee (Ethics approval number 1000000703). Approvals were also obtained from the Queensland Department of Education and Training and Employment (DETE) and the University of Sydney Human Research Ethics Committee.

### 2.3. Selection of Schools

Government-run primary schools were selected from the Brisbane Metropolitan Area using the following selection criteria:

(1) The school had naturally ventilated classrooms used by eight to eleven year old students.
(2) There were no major road constructions, infrastructure projects, or building works planned in the vicinity of the school during the study period.
(3) There were no major local air pollution sources, other than vehicular traffic, close to the school. Schools that fulfilled the selection criteria were invited to participate.

### 2.4. Selection of Classes and Children

We have chosen children as a population because children are generally more susceptible to air pollution and their attendance at school for six to seven hours a day during weekdays allows us to assess the impact of their pollution exposure at school. 

A number of classrooms that included students in years three, four and five were selected at each participating school. The number of classrooms included from each school depended on the number of students within the eligible age group (8 to 11 years) in each classroom. All eligible children in the selected classes were invited to participate. 

### 2.5. Recruitment of Participants

Between 3 and 9 weeks before the scheduled date for testing participants, we introduced the study to the students through a 10 min presentation with an opportunity for questions. We then gave each student a take home information pack. The packs included a written information sheet for parents and one in simpler language for the students, two copies of the consent form, and a questionnaire. In some schools, we re-introduced students in the participating classrooms to the project on the first day of field testing. Only students whose parent or guardian completed, signed and returned the consent form were tested.

### 2.6. Ultrafine Particles from Traffic Emissions and Children’s Health (UPTECH) Questionnaire

The questionnaire was developed based on questions from the International Study of Asthma and Allergies in Childhood (ISAAC) questionnaire (ISAAC Steering Committee and ISAAC Phase Three Study Group, 2000) [10] the Belmont Schoolchildren Study [11] and the Childhood Asthma Prevention Study questionnaire (CAPS) [12]. The questionnaire collected information about respiratory symptoms and illnesses, general health status and parental history of respiratory disease, as well as potential confounders and effect modifiers relevant to the analysis. These included:

- Housing conditions; such as the type of home and garage, the presence of pets, and whether or not there is carpeting;
- Home environment; such as occurrence of flooding or water damage, presence of mould, mildew or a musty odour;
- Socio-economic status through both parent’s highest level of education;
- Other sources of exposure to air pollution, including fuels used for cooking and heating at home and exposure to traffic during commuting on weekdays and on weekends;
- Tobacco smoke exposure and;
- Ethnicity.

Home addresses and information about travel times and routes between home and school were also collected to assess exposure to UFPs outside the schools. The questionnaire was pilot tested to validate new components, especially regarding UFP exposure, as well as logical flow, feasibility and appropriateness of wording [13].

### 2.7. Clinical Measurements

Clinical testing was conducted over a one week period at each school during one of the two weeks when air quality measurements were conducted. All participants underwent a series of tests to assess airway function (including peripheral airway function), airway inflammation, allergic status, exposure to second hand tobacco smoke and systemic inflammation. Blood was collected for subsequent DNA extraction and genetic analysis. Participants were able to opt out of blood tests (including the genetic tests) and allergy skin prick tests while still participating in the other tests. Standing height was measured using a stadiometer and weight was measured using bathroom scales. The total time taken to complete all the health tests was approximately two and a half hours per child. All equipment was calibrated daily.

#### 2.7.1. Spirometry

Spirometric lung function was measured, before and after the administration of a bronchodilator, using a Spirocard device (QRS Diagnostic, LLC, Plymouth, MN, USA) linked to a portable computer running SpiroScore+ V2.6 software (Bird Healthcare, Melbourne, Australia) in accordance with ATS/ERS recommendations [14]. The tests were conducted by highly trained technicians; the collected data was checked for quality after that. Short-acting bronchodilators were withheld for six hours and long-acting bronchodilators for 12 h prior to the testing session. The procedure was repeated three or more times, until the difference between the best and the second best values for FEV_1_ was less than 100 mL. Immediately after the baseline spirometry was completed, we administered salbutamol 200 µg using an MDI device (Ventolin^®^ 100 µg) via a tube spacer. Post-bronchodilator measurements were made 10 min later using the same procedure. The post-bronchodilator spirometry was the last test each child performed on the test day to avoid any effects of salbutamol on the other breathing tests.

#### 2.7.2. Fraction of Exhaled Nitric Oxide (FeNO)

Exhaled nitric oxide was measured as a marker of airway inflammation. Samples of exhaled breath were collected into sealed inert bags at a flow rate between 10 and 14 litres per minute using a rotameter built at the Woolcock Institute of Medical Research (WIMR). Several breaths were usually required to obtain an adequate volume that could be analysed offline. The exhaled gas was analysed within six hours of collection using a chemiluminescence analyser (Thermo Environmental Instruments, Lear Siegler, Australia). Measurement of FeNO was the first test conducted as it is possible for other breathing tests, such as spirometry and MBNW, to have an effect on natural FeNO levels.

#### 2.7.3. Forced Oscillation Technique (FOT)

FOT provides a tidal breathing-based measurement of airway mechanics. Respiratory system impedance was measured at 6 Hz as this allows assessment of peripheral airways mechanics. Measurements were performed using an in-house FOT device, as described in detail previously [15], but modified to reduce total equipment dead space to comply with recent paediatric recommendations [16]. Impedance repeatability checks and volume checks were performed at the start and end of each testing session. At each visit, following a practice test, a series of technically acceptable one minute FOT measurements were performed, with the child sitting upright, wearing a nose clip, and with cheeks and floor of mouth supported (by the child under instruction). Children watched a DVD during the test to encourage regular and comfortable breathing. Recordings were deemed acceptable by the technician if tidal volume and breathing frequency appeared stable, with no obvious leaks or glottic closures identified by visual inspection of the volume trace. Breath-by-breath data filtering was used to identify and reject entire breaths in which respiratory artefact occurred [17]. Rrs and Xrs was recorded for each session as the mean of all acceptable tests. The results were expressed as raw values due to the lack of equipment-specific FOT normative data in this age group.

#### 2.7.4. Multiple Breath Nitrogen Washout (MBNW)

MBNW is a tidal breathing tests which measures peripheral airway function by assessing the gas mixing efficiency within the lungs [18]. The peripheral airways are the predominant site of gas mixing due to their large surface area. Children performed the test connected to a mouthpiece containing a bacterial filter, and wore a nose clip. Children watched a DVD during testing for distraction purposes, to encourage a regular breathing pattern. During the wash-out portion of the test, children were switched to breathing 100% oxygen until the end-tidal nitrogen concentration (approximately 78%) had been washed out to below 1/40th of the starting end-tidal concentration (approximately 2%). The test was performed using a custom built MBNW device built (WIMR, Sydney). Flow was measured using a Hans Rudolph pneumotachograph (3700 series, flow range 0–160 L/min, Hans Rudolph, Vacumed, Ventura, CA, USA). Nitrogen concentrations were measured using a Medgraphics nitrogen analyser. The accuracy of the MBNW system had been validated against a range of known lung model volumes, consistent with the approach taken by other research groups at the time of the pilot study [19]. A minimum of four technically acceptable washouts were attempted with each child. The number of washouts per child was decided based on the pilot study (results presented below). Between washout runs an interval of double the washout time was used to allow end-tidal nitrogen concentration to return to baseline concentration (approximately 78%).

#### 2.7.5. Allergy Skin Prick Tests

We measured atopic status by skin prick test using the following environmental airborne allergens: house dust mites (*Dermatophagoides pteronyssinus* and *Dermatophagoides farinae*), cockroach; cat; two moulds (*Alternaria* and *Aspergillus*); rye grass and a grass mix (Hollister-Stier, Spokane, WA, USA). Glycerol and histamine phosphate 10 mg/mL were used as negative and positive controls, respectively. Wheal sizes were measured 15 min after the tests were performed [20]. The average wheal size was calculated as the mean of the longest transverse diameter and its perpendicular. Wheals that were ≥3 mm and were also >the negative control were considered positive.

#### 2.7.6. Blood Tests

Blood was collected by trained phlebotomists after using an EMLA^®^ dermal anaesthetic patch. Highly sensitive C-reactive protein (hsCRP) (Tina-quant C-reactive protein Gen.3, Roche Diagnostics, Indianapolis, IN, USA) and cotinine [21] were measured in serum as indicators of systemic inflammation and recent exposure to environmental tobacco smoke, respectively. DNA extracted from whole blood for later genomic analysis.

### 2.8. Air Quality and Particle Exposure Measurements

Air quality measurements were performed continuously for two consecutive weeks at each school. UFP particle number concentration (PNC) was measured at three outdoor sites within the grounds of each school. These sites were selected in order to describe the exposure profile with respect to traffic conditions and prevailing wind direction. Two outdoor sites were located at the two ends of the school grounds downwind of the prevailing wind direction and a third outdoor site was located in a central location on the axis.

In addition to PNC, other air quality parameters were measured at one of the centrally located outdoor sites. These included particle mass in various fractions (PM_1_, PM_2.5_, PM_4_ and PM_10_), particle surface area, particle number size distribution, elemental and organic carbon (EC and OC) in PM_2.5_, elemental composition of PM_2.5_ and PM_1_, volatile organic compounds (VOCs), polycyclic aromatic hydrocarbons (PAHs); carbonyls, gases (oxides of nitrogen, carbon monoxide, carbon dioxide and sulfur dioxide), pollen, fungi, ions and meteorological parameters (temperature, humidity, wind speed, and direction). A Compact Time-of-Flight Aerosol Mass Spectrometer (C-ToF-AMS, Aerodyne, MA, USA) was deployed to examine spatial and temporal variability of organic aerosols in the outdoor air, and positive matrix factorization (PMF) was used to apportion the sources of the organic aerosols across the Brisbane Metropolitan Area (for the first time in the Southern Hemisphere).

PNC, carbon dioxide, polybrominated diphenyl ethers in the dust, chemicals (VOCs and carbonyls), and fungi, endotoxin concentrations and temperature were measured in the two selected naturally ventilated classrooms, at the same time as the outdoor monitoring. 

We used structured surveys to record information on classroom characteristics (e.g., number of occupants, size and type of flooring), daily activities, potential sources of indoor particles (and their operation schedule) as well as classroom cleaning schedules. Traffic density was recorded for the busiest road adjacent to the school. Details on the indoor and outdoor air quality monitoring, methodologies, instrument and data quality control checks and outcomes have been published [22,23,24,25,26]. 

At each school three to six of the participating children undertook personal UFP exposure monitoring for 24 h with the consent of their teacher and parent or guardian. The methodology and outcomes of this study have been recently published [26].

PNC were continuously measured at three long-term urban reference sites. These sites were: Queensland University of Technology (Gardens Point Campus) (1 January 2009–31 December 2009 and 20 September 2010–continuing), and two QLD Department of Environment and Heritage Protection (DEHP) air quality monitoring stations in Rocklea (1 January 2009–31 December 2009) and Woolloongabba (1 January 2009–31 August 2010). Using data from the reference sites and 25 schools, a long term time series model has been developed to examine the relationship between PNC concentrations and meteorology at both site-specific and airshed scales [27].

### 2.9. Data Management

Microsoft Access databases were used to collate and store the air quality, questionnaire and clinical test data. As a quality control check, written questionnaire data was double entered by separate data entry staff. Data entries that did not match were compared against the original hard copies and corrected. Up to five attempts were made to contact parents by phone in order to complete missing data in questionnaires. 

All the spirometry data was checked for repeatability and acceptance against the American Thoracic Society (ATS) criteria using in-house quality control software. All MBNW and FOT data were checked for quality within the Woolcock Institute of Medical Research.

### 2.10. Data Analysis

#### 2.10.1. Analysis Plan

A function will be fitted to describe the distribution of particle number concentration (the main exposure of interest) at each school, either as a single log-normal density, a mixture of log-normals or a histogram. A Bayesian hierarchical linear model will then be fitted to assess the relation between this exposure distribution function and each of the main clinical outcomes to be tested: asthma diagnosis, respiratory symptoms, spirometric lung function, and exhaled nitric oxide. Functions representing the distribution of measured concentrations of other pollutants at the school site and home environmental factors that were assessed by questionnaire will be included as potentially confounding covariates.

The Bayesian hierarchical model is a Generalised Linear Mixed Model with a two-level hierarchy: classrooms within schools, schools within the study area. The use of exchangeable priors at each level of the hierarchy allows for partial pooling of the data [28]. This partial pooling offers a compromise between complete pooling (treating class-room level variables as fixed effects in the model) and treating each class as an independent cohort within the study. Whether the effects of explanatory variables and confounders are linear will be investigated by making use of semi-parametric regression methods that allow for flexible non-linear effects without specifying the non-linear form *a priori* [29].

Model building will be based on a conceptual model that incorporates previously studied relationships between air quality and health, with the potential confounder variables being the age, sex, atopic status, asthma diagnosis, *etc.* at the individual level, as well as characteristics of the home that are known to explain variation in the clinical outcomes. A linear partial effect for each of the explanatory variables will be fitted in order to assess whether each explanatory variable has a positive, negative or neutral effect on the health outcome variable of interest. Bayesian model choice will be by Deviance Information Criterion, a goodness of fit parameter that accounts for the number of parameters that are included in the model. As most of the confounders (e.g., sex, atopic status, home characteristics and health history) are binary variables, the effect sizes will be directly comparable for determining which confounders are most important modifiers of the exposure-response relationship.

#### 2.10.2. Sample Size and Study Power

The sample size and power calculations were based on measuring between-subject differences in FEV_1_ and in the prevalence of asthma. The calculations were performed in PASS 2008 software (NCSS, UT, USA). The between-subject standard deviation in children, which was estimated from the recently conducted Australian Child Health and Air Pollution Study (ACHAPS), was assumed to be 225 mL [30]. The mean baseline FEV_1_ was assumed to be 2000 mL. The intra-cluster (that is, intra-school) correlation coefficient, estimated from the same study, was 0.03. The baseline prevalence of asthma was assumed to be 15% [31]. We estimated that a sample size of 343 would yield 80% power to detect a significant effect at the 5% level for: a difference in FEV_1_ attributable to a 1 standard deviation change in pollutant exposure of 109 mL (or 5.4% of baseline); a difference in FEV_1_ attributable to 2 standard deviation change in pollutant exposure of 54 mL (or 2.7% of baseline); and a difference in FEV_1_ attributable to a change in pollutant exposure from the 5th to the 95th percentile of 33 mL (1.7% of baseline). This sample size would also give 80% power to detect an odds ratio for the risk of asthma associated with a 1 standard deviation change in pollutant exposure equal to 1.6 or greater. As the subjects were clustered within schools, it was necessary to adjust this sample size for the effect of clustering. Assuming that 30 subjects would be recruited per school, then the design effect for clustering would be 1.87. This yielded a final sample size of 641 subjects recruited from 21 schools (clusters). To allow for potential loss to follow-up, we decided to recruit 25 schools. Based on an expected response rate of 30%, we approached 99 students aged 8 to 11 from each school to join the study.

### 2.11. Pilot Study

In March (autumn) 2009, a pilot study was conducted prior to the main UPTECH study to test the feasibility and the reproducibility of the clinical measurements that had not previously been implemented in field studies. Based on the pilot study we were able to determine which outcome measures to include and how many repeated measurements should be made for each test. The pilot study did not include air quality measurements.

Forty eight children, aged between 8–11 years, were selected from two primary schools in the Brisbane area. This number was based on the estimated total time taken to complete the testing protocol for each child (1 h), the number of children who could be tested simultaneously and the time available in a normal school day (8:00 A.M. to 3:00 P.M.). Questionnaires that were completed by parents as part of the consent process provided information on previous and current respiratory health of the children. Children consenting to participate were tested during school hours. 

The same testing protocol was repeated two weeks later with the tests performed at the same time of day as each participant’s first visit, in the same school setting. 

All the clinical tests, except measurement of FeNO, were conducted using the methods described above. No blood specimens were collected in the pilot study. In the pilot study, FeNO was measured using the Hypair (Medisoft^®^, Sorinnes, Belgium) device in parts per billion (ppb). This device measures FeNO using an electrochemical cell NO analyser within the device. The test was performed, in duplicate, at three expiratory flow rates (50, 100, and 150 mL/s). Incentive software was used to encourage children to maintain the desired flow rate for the exhalation period of 3 s after the flushing of equipment-related dead space. Children performed trial runs at each flow rate until the technique was deemed adequate by the operator. For each child, the NO output (exhaled NO multiplied by flow rate, *y*-axis) was plotted against flow (*x*-axis) for each of the six NO measurements. A first order linear regression across the data points was performed. Alveolar NO (Calv, ppb), an index of inflammation in the peripheral airways [32], was calculated as the slope of this regression line and bronchial flux (JNO, pL/s), was calculated as its y-axis intercept. 

The reliability of all of the clinical tests was assessed by calculating the intraclass correlation coefficient (ICC) using data from the two testing sessions. ICC is expressed on a scale of 0 to 1, with 1 representing perfect reliability. 

## 3. Results of Pilot Study and Discussion

### 3.1. Pilot Study Results 

The demographic and clinical characteristics of the 48 children recruited for the pilot study, 46 (96%) of whom completed the second testing session, are shown in Table 1. Eleven of the 48 (23%) children had a history of doctor-diagnosed asthma, as reported in the parent-completed questionnaire and 18 of 42 children (43%) were atopic, based on the results of skin prick testing. The prevalence of atopy in the pilot study population is similar to the reported prevalence in other Australian populations [33] and the prevalence of asthma in this study population is also similar to the reported prevalence of ever diagnosed asthma in children aged 0 to 15 years, which ranged between 13% and 25% across different states in Australia. [34]. At the time of testing all of the children were able to successfully complete duplicate FeNO measurements at each of the three flow rates. On further examination of the resulting flow and pressure profiles, 254 of 264 (96.2%) individual FeNO measurements were deemed to be satisfactory. Ten values were rejected due to a fall in pressure to zero during the collection period. Six of these occurred at 50 mL/s, one at 100 mL/s, and three at 150 mL/s. Six data points were available for calculation of Calv and JNO in 43 of 48 (89.6%) at the first visit and 44 of 46 (95.7%) at the second visit, with five available for 4 of 48 (8.3%) and 2 of 46 (4.3%) respectively. The remaining children from the first visit had four data points with at least one data point for each flow rate. The average of two acceptable results from each week was taken as the value for calculation of between-session repeatability. If only one acceptable value for the visit was available then no average value was used for that visit. The ICCs for Calv and JNO were 0.37 and 0.87, respectively. Although the same measurements have been conducted in previous studies using school children [35], these data show that alveolar NO (Calv) was not measured reliably enough for application in field (or clinical) studies using this technique. 

**Table 1:** Demographic and clinical characteristics of children recruited for the Ultrafine Particles from Traffic Emissions and Children’s Health (UPTECH) pilot study (*N* = 48).

Characteristic	Mean (SD) or Number (%)
Age (years)	9.27 (0.89)
Sex (Male)	21 (44%)
Height (cm)	137.42 (6.47)
Height (percentile)	50.02 (24.27)
Height (*z*-score)	0.00 (0.70)
Weight (kg)	33.56 (6.73)
Weight (percentile)	53.63 (25.48)
Weight (*z*-score)	0.13 (0.81)
Atopic (*N*, %)	18/42 (43%)
Doctor diagnosed asthma (*N*, %)	11 (23%)

Acceptable FOT measurements from both visits were obtained in 43 of the 48 children. Three were rejected on analysis (one due to tachypnoea on the first visit and two due to the presence of frequent artefacts or variable leaks on the second visit). There was no significant difference in Rrs6 or Xrs6 values between participants with and without asthma. The ICCs for Rrs6 and Xrs6 are shown in Table 2. Both Rrs6 and Xrs6 were highly reproducible in these participants.

**Table 2:** Reliability of respiratory tests conducted in the UPTECH pilot cohort.

Respiratory Test	Intraclass Correlation Coefficient (95% Confidence Interval)
FeNO (*n* = 46)	
JNO (intercept)	0.87 (0.78–0.93)
Calv (slope)	0.37 (0.10–0.59)
FOT (*n* = 43)	
Rrs	0.81 (0.68–0.89)
Xrs	0.81 (0.67–0.89)
MBNW (*n* = 45)	
LCI	0.64 (0.43–0.78)
Spirometry ^*^ (pre *n* = 46) (post *n* = 43)	
FEV_1_ pre	0.93 (0.88–0.96)
FEV_1_ post	0.90 (0.83–0.95)
FVC pre	0.92 (0.85–0.95)
FVC post	0.94 (0.90–0.97)

^*^ Pre- = pre-bronchodilator, post = post-bronchodilator.

All children completed MBNW testing. Two of the 48 children on the first visit did not produce acceptable results due to poor technique (e.g., tachypnoea that did not respond to distraction during testing). Acceptable paired results were obtained in 45 of the 46 (97.8%) children who attended both sessions. The ICC for LCI was 0.64 for the whole cohort, indicating adequate reliability. Spirometric measurements were also highly reliable. Only 43 out of 46 children completed the post bronchodilator test. ICCs for pre- and post-bronchodilator FEV_1_ and FVC were between 0.92 and 0.94.

### 3.2. Discussion

The UPTECH study is the first large multidisciplinary epidemiological study to investigate respiratory health effects associated with children’s exposure to traffic-related UFP emissions.

The pilot study demonstrated the feasibility and, except for alveolar NO, the reliability of the proposed clinical measurements. As a result of the pilot study it was decided not to include the alveolar NO measurements in the main study and revert to measuring total expired NO (FeNO) using the rotameter method, which is simpler and has been shown to be reliable in several previous studies [11,36,37]. 

One of the strengths of this study design is that it enables us to link exposures at schools with health endpoints and covariates measured on individual students attending that school. We have already demonstrated that there is a wide range of UFP number concentration and traffic densities among the schools selected for this study [24]. This will improve the power of the study to detect health effects attributable to this exposure. The broad range and long duration of continuous air pollution measurements (2 weeks), as well as the multiple indoor and outdoor measurement locations within each school, allowed more accurate estimation of children’s exposure within the school environment. 

A further strength of the study is the use of FOT and MBNW to assess the peripheral airways, which is the area of the lung most likely to be affected by UFP. There has only been one study looking at the effects on UFP on mid-flow spirometry (FEF25-75). That study found an association between UFP exposure and the level of FEF25-75 [38]. The peripheral airways measurements that are being conducted in the UPTECH study are novel and will complement information on respiratory symptoms and diagnoses, as well as well-established spirometric lung function measures and measures of airway inflammation. 

FOT has been previously measured in a field setting [39]. However this is the first time, to our knowledge, that MBNW measurements have been conducted in a field setting. The pilot study has shown that the spirometric measurements were more repeatable; however the FOT and MBNW are a more sensitive measure for peripheral airway abnormalities. These data will provide us with valuable information regarding the effects of air pollution on peripheral airways.

The study has been adequately powered to detect meaningful health effects. A broad range of potential confounders have been measured. The other strength of this study was personal particle number exposure monitoring. Although personal exposure monitoring was limited to small number of participating children, due to a limited number of available instruments and time constraints, it allowed the assessment of children’s personal UFP exposure at school, in the home, while commuting and in other non-school environments. 

In the main UPTECH study we collected data on air pollution and on clinical endpoints all year round, except during school holidays. Hence both seasonal and regional variation in climate might contribute to observed variation in clinical outcomes [40,41,42]. For this reason, we have also collected relevant meteorological data that will allow us to test the effects of seasonality and temperature changes on clinical outcomes.

The study does have a number of important limitations that will need to be considered in interpreting the findings. The cross sectional design of the study, means that incident events cannot be quantified and it may be difficult to separate spatial (school-level) and temporal (date of measurement) sources of variation. We believe this initial cross-study may lead to future cohort studies to address this issue. Furthermore, for the main analyses indoor exposure status was considered to be the same for all participants in the same classroom and outdoor exposure status was considered to be same for all participants at the same school. The main variables for exposure-response analysis were not assessed at an individual level. This is most relevant to our study objective, which is to assess the impact of school-based environmental exposures on respiratory health outcomes. 

## 4. Conclusions 

We have shown that it is feasible to conduct comprehensive peripheral airway testing on children in the school setting. These tests are invaluable for assessing the potential respiratory health impact of UFP exposure in children. To the best of our knowledge this is the first study to assess the effects of traffic-derived UFPs on peripheral airways. The extensive exposure and health data collected for the UPTECH study, as well as the multiple sites where the study was conducted, will help give us a clearer picture on the effects of UFP on human health. Besides showing the feasibility of the tests being conducted and highlighting the results from the pilot study, this paper describes the details of the main UPTECH study and can be referenced in subsequent results manuscripts. 
